# Population Genetics of *Schistosoma japonicum* within the Philippines Suggest High Levels of Transmission between Humans and Dogs

**DOI:** 10.1371/journal.pntd.0000340

**Published:** 2008-11-25

**Authors:** James W. Rudge, Hélène Carabin, Ernesto Balolong, Veronica Tallo, Jaya Shrivastava, Da-Bing Lu, María-Gloria Basáñez, Remigio Olveda, Stephen T. McGarvey, Joanne P. Webster

**Affiliations:** 1 Department of Infectious Disease Epidemiology, Faculty of Medicine, Imperial College London, London, United Kingdom; 2 Department of Biostatistics and Epidemiology, College of Public Health, Health Sciences Center, University of Oklahoma, Oklahoma City, Oklahoma, United States of America; 3 Research Institute for Tropical Medicine, Muntinlupa, Philippines; 4 Ateneo de Manila University, Quezon City, Philippines; 5 International Health Institute, Brown University, Providence, Rhode Island, United States of America; Biomedical Research Institute, United States of America

## Abstract

**Background:**

*Schistosoma japonicum*, which remains a major public health problem in the Philippines and mainland China, is the only schistosome species for which zoonotic transmission is considered important. While bovines are suspected as the main zoonotic reservoir in parts of China, the relative contributions of various non-human mammals to *S. japonicum* transmission in the Philippines remain to be determined. We examined the population genetics of *S. japonicum* in the Philippines in order to elucidate transmission patterns across host species and geographic areas.

**Methodology/Principal Findings:**

*S. japonicum* miracidia (hatched from eggs within fecal samples) from humans, dogs, pigs and rats, and cercariae shed from snail-intermediate hosts, were collected across two geographic areas of Samar Province. Individual isolates were then genotyped using seven multiplexed microsatellite loci. Wright's *F_ST_* values and phylogenetic trees calculated for parasite populations suggest a high frequency of parasite gene-flow across definitive host species, particularly between dogs and humans. Parasite genetic differentiation between areas was not evident at the definitive host level, possibly suggesting frequent import and export of infections between villages, although there was some evidence of geographic structuring at the snail–intermediate host level.

**Conclusions/Significance:**

These results suggest very high levels of transmission across host species, and indicate that the role of dogs should be considered when planning control programs. Furthermore, a regional approach to treatment programs is recommended where human migration is extensive.

## Author Summary

Schistosomiasis is a disease caused by parasitic worms known as schistosomes, which infect about 200 million people worldwide. In the Philippines, as in China, the species of schistosome (*Schistosoma japonicum*) which causes the disease infects not only humans, but also many other species of mammals. In China, bovines are thought to be particularly important for harboring and transmitting *S. japonicum*, whereas in the Philippines infections in bovines are relatively rare. However, dogs, rats and pigs are often infected with *S. japonicum* in the Philippines, although the extent to which infections in these animals may give rise to human infections is unclear. To help answer this question, we characterized the genetic variation of the parasite in Samar province of the Philippines, and found that *S. japonicum* samples from humans, dogs, rats and pigs were genetically very similar, with no significant genetic difference between samples from humans and dogs. This suggests that in the Philippines this parasite is frequently transmitted between different mammalian species, particularly between dogs and humans. Reducing levels of infections in dogs may therefore help to reduce infections in humans. The results also suggest high levels of transmission between geographic areas, thus regional co-ordination of treatment programs is recommended.

## Introduction

Infection by the Asian blood fluke *Schistosoma japonicum* remains an important public health burden in the Philippines, China and parts of Indonesia, despite continued efforts of ongoing control programs [1]. In the Philippines alone, it has been estimated that approximately 6.7 million people live in areas endemic for *S. japonicum* [2]. *S. japonicum* is unique among the species of schistosomes infecting humans, in that it can infect more than 40 other species of mammalian hosts and is the only species for which zoonotic transmission is considered important [3]. Recent studies by our group in Samar, the Philippines, found high prevalence and intensities of *S. japonicum* infection in dogs and rodents [4], with intensities of infections in dogs at the village level found to be associated with the intensity of human infection [5]. Conversely, the same data used in a transmission dynamics model suggest that rats may play some role in human infection [6]. However, from such parasitological data alone it remains unclear which, if any, of these animals are most important as zoonotic reservoirs for human infection.

Molecular tools are increasingly being applied to address questions concerning parasite epidemiology [7], and the usefulness of molecular markers as tools for studying the transmission and host-specificity of parasites has been recently highlighted [8]. For example, molecular studies of *Ascaris* nematode populations in pigs (*A. suum*) and humans (*A. lumbricoides*) suggest there is significant genetic subdivision between the two parasite populations [9], although some cross transmission may occur between pigs and humans in sympatric locations [10],[11]. Unfortunately the number of similar molecular epidemiological studies on multi-host parasites such as *S. japonicum* is limited.

Several researchers have called for a better understanding of schistosome genetic diversity and structure, and have recommended the use of microsatellite markers for such studies [12],[13],[14]. Microsatellite markers isolated and characterized for *S. japonicum* have shown significant polymorphism between isolates, making them highly useful for studying the population genetic structure of the parasite [15]. A recent study by Wang et al. [16] employed these markers to investigate population genetic structure in relation to definitive host species in the marshland region of Anhui province, China, and found that *S. japonicum* larval samples segregated into two main groups: isolates from humans, cattle and water buffalo clustered together, while isolates from dogs, cats, goats and pigs formed a second cluster. This suggested that human transmission in this region of China is more closely associated with bovines than with other domesticated animals. These markers have also revealed structuring of *S. japonicum* genotypes in China according to geographic region (mountainous vs. lake/marshland areas) and intermediate host morphology (ribbed- vs. smooth-shelled *Oncomelania hupensis* snails), and also between China and the Philippines [17].

In the present study, we obtained *S. japonicum* larval isolates from four definitive host species and snail-intermediate hosts from two geographical areas within Samar Province of the Philippines. Genetic analysis of schistosome larvae sampled directly from naturally infected hosts (miracidia hatched from eggs excreted by infected mammalian hosts, and cercariae shed from infected snail-intermediate hosts) is now feasible, and eliminates the need to passage schistosomes through laboratory hosts [18]. This is particularly advantageous for molecular studies of *S. japonicum*, which shows strong bottlenecking of genotypes following passage through rodents [19]. In addition, the molecular techniques used in the aforementioned studies by Shrivastava et al. [17] and Wang et al. [16] have been further developed to allow multiple loci to be genotyped for each larval isolate, allowing for more robust genetic analyses [20].

Through application of these novel techniques, we present here the first population genetic study of *S. japonicum* in the Philippines, in which the main aims were to assess levels of parasite gene-flow, and thus also transmission, across different host species and geographic areas in the Philippines. A comparison is also made with previous findings in China, where the epidemiological setting for *S. japonicum* differs considerably from the Philippines in terms of the suspected animal reservoirs, snail hosts, topography, and seasonality of transmission. Implications in terms of potential differences in the evolution of *S. japonicum* between the two countries, and applications for current targeted control activities, are discussed.

## Materials and Methods

### Study sites

**Figure 1:**
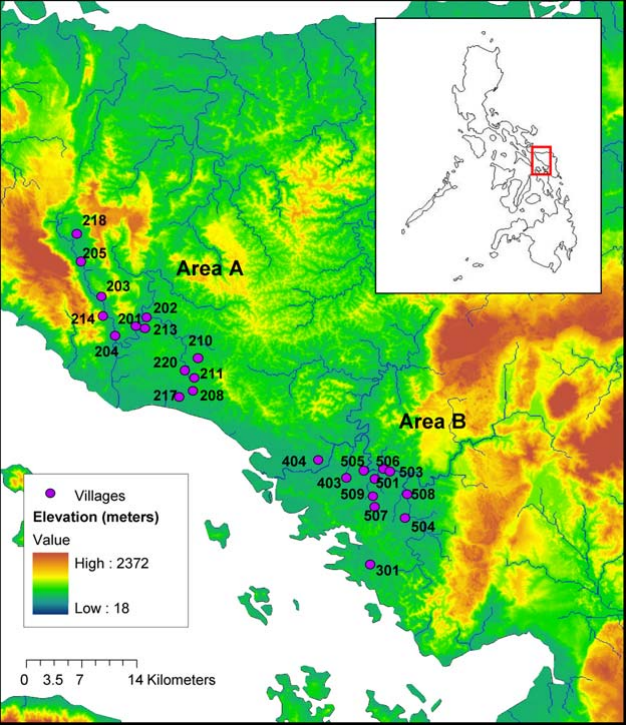
Location of the villages sampled in two geographic areas (A and B) of Samar Province, the Philippines. The inset map shows the location of Samar in the Philippines. Adapted from an original version in Akullian A (2007) Spatial patterns of schistosomiasis: A watershed approach to measuring *S. japonicum* environmental risk and human and animal disease outcomes [Sc.B. Thesis]. Brown University.

The molecular analysis was a subcomponent of a larger epidemiological study called Schistosomiasis Transmission and Ecology in the Philippines (STEP). The objective of the STEP study was to estimate the effect of man-made irrigation on human infection with schistosomiasis japonica in Samar province, region of the eastern Visayas, in the Philippines. One major specific aim was to estimate the role of infection in five animal species (cats, dogs, pigs, rats and water buffaloes) in human infection. The main study took place over 18 months in 50 villages (known locally as ‘barangays’), selected according to the number of hectares of farmland under water management and irrigation systems. Further details of the selection of villages can be found elsewhere [5],[21]. During the statistical analysis of the data, the prevalence of infection in humans and animals was found to heavily cluster within three geographical areas in the province (referred to as areas A, B and C) [21]. Sampling for the molecular analysis was conducted from May to December 2005 across all three areas. However, in area C only a relatively small sample of isolates was obtained, which was unlikely to be representative of the genetic diversity within this region and was therefore excluded from the analysis. This paper therefore reports on samples collected across 24 villages in areas A and B. In area A, villages were scattered along narrow river valleys within an area of approximately 136 km^2^, while in area B villages were situated among open foothills within an area of approximately 127 km^2^ [21]. Villages within each area were highly interconnected by watersheds. A map of the study region is shown in Figure 1.

### Sampling from definitive hosts

Details on the selection, sampling, and measurement of infection status of human participants and animals for the STEP study can be found in [5] and [4], respectively. Briefly, up to six humans from up to 35 randomly selected households in each village were asked to provide stool samples over three consecutive days which were subjected to Kato-Katz examination. Due to financial and logistical constraints on the molecular subcomponent of the STEP study, only a proportion of individuals testing positive in the parasitological surveys were retraced to obtain the molecular data presented here. To collect stools from non-human animals, dogs were tethered and cats and puppies were placed in cages (supplied with food and water) and allowed to defecate. Per rectal sampling was carried out on pigs and water buffaloes (carabao). Stool samples were collected for two consecutive days in animals, with the same animals sampled on both days. In each village, 30 rat traps were set for three days, and fecal samples were collected from the floor of the cage. The Danish Bilharziasis Laboratory (DBL) sedimentation technique was used to determine the infection status of non-human animals [22],[23].

Where possible, miracidia were hatched from infected fecal samples according to standard protocols [24],[25]. In order to minimize contamination, miracidia were washed individually by sequential transfer to three successive Petri dishes containing autoclaved deionized water, and their DNA was stored on Whatman FTA cards according to protocols described elsewhere [18],[19]. The number of hosts and miracidia isolates sampled across host species and geographic areas that were successfully genotyped are summarized in Table 1, where it can be seen that the majority of isolates were obtained from humans and dogs (21 and 17 individuals, respectively). Isolates were also obtained from 4 rats and 3 pigs, whilst no miracidia were successfully hatched from cats or water buffaloes.

**Table 1:** Number of *Schistosoma japonicum* isolates genotyped and number of hosts sampled by geographic area and host species.

Geographic Area	No. of miracidia (No. of hosts sampled)				No. of cercariae (No. of unique cercariae genotypes)	Total no. of isolates
	Dog	Human	Pig	Rat		
A	138 (7)	39 (2)	42 (3)	0 (0)	141 (57)	360
B	190 (10)	370 (19)	0 (0)	42 (4)	236 (93)	838
Total	328 (17)	409 (21)	42 (3)	42 (4)	377 (150)	1198

### Sampling from snail-intermediate hosts

Snail survey sites within villages were selected based on the types of aquatic environments most likely to support *Oncomelania quadrasi* [26], which is the snail-intermediate host for *S. japonicum* in the Philippines [27]. Well-shaded areas along streams, springs or various canals (drainage canals and others) and swampy areas or grass land (often currently unplanted rice fields) were inspected for the presence of snail colonies. All collected *O. quadrasi* were transported to the laboratory and checked for shedding of cercariae using standard protocols [25]. Due to the low prevalence of infection among snails and time constraints in the field, all snails collected within each village were pooled together when examining for cercarial shedding, thus cercarial isolates could not be assigned to individual infected snails. However, to prevent any potential bias caused by sampling multiple clones arising from a single snail, the cercariae dataset was cleared of all clonal genotypes within each village prior to analyses (see below). As with miracidia, cercariae were washed in autoclaved deionized water and stored on Whatman FTA cards [18]. The number of cercariae genotyped from each geographic area is shown in Table 1.

### Genotyping schistosome DNA

Of the 11 previously isolated and characterized *S. japonicum* microsatellite markers [15], seven were used in this study, namely MPA, RRPS, TS2, J5, 2AAA, M5A and MF1. The other four markers were excluded due to high frequency of null alleles or poor amplification in multiplex reaction conditions (as distinct from the single locus reactions used in the previous studies [16],[17]), despite considerable time taken to optimize reaction conditions during assay development. Primers were checked for interactions using Autodimer software [28]. The 5′ end of the forward primer for each locus was fluorescently labeled using either 6-FAM, VIC, PET or NED (Applied Biosystems), using different colors for loci with overlapping size ranges.

Larval (miracidia and cercariae) samples were removed from the Whatman FTA cards using a Harris Micropunch, and placed individually in 96-well dishes. Samples were washed in FTA Purification reagent and Tris-EDTA (TE) buffer according to standard protocol [18], before adding to each well 25 µl of reagent mix, containing 0.01 µM of primers (0.005 µM for RRPS, M5A and J5), 3 mM MgCl_2_, ultra-pure high quality dNTP Mix and HotStarTaq DNA polymerase (Qiagen Multiplex PCR kit, West Sussex, UK). Polymerase chain reaction (PCR) amplifications were performed on a PTC-200 Thermal Cycler (MJ Research), using a stepdown PCR beginning with an initial hot-start activation at 95°C for 15 min, followed by 40 cycles of 30 sec at 94°C, 90 sec at annealing temperature (2 cycles at each temperature from 60°C to 56°C, then 20 cycles at 55°C), and 60 sec at 72°C, with a final extension at 60°C for 30 min. 2 µl of PCR product, along with LIZ-600 size standard (Applied Biosystems) was subjected to electrophoresis using an ABI Prism 3730 Genetic Analyzer (carried out by Geneservice, Oxford, UK). Allele sizes were assigned using Genemapper version 4.0 (Applied Biosystems).

DNA from one of five *S. japonicum* adult worm isolates from China were genotyped on each PCR plate to check for consistency of marker amplification across PCR runs, and also consistency of allele calling across genotyping runs. Details on the origin and DNA extraction of these adult worm isolates can be found in [17]. Cercarial isolates from three villages (Guanghui, Heping and Xingzhuang) from a marshland region of Anhui Province, China, collected in March–April 2006, were also genotyped as a control group. This was to confirm that the microsatellite markers could differentiate between isolates from quite separate and isolated regions, between which one would expect there to be little, if any, parasite gene-flow.

### Data analyses

The number of alleles, gene diversity (unbiased expected heterozygosity), and polymorphism information content (PIC) values were calculated using PowerMarker version 3.25 [29]. Genetic diversity was also characterized by calculating allelic richness in FSTAT version 2.9.3.2 [30], as this accounts for differences in sample sizes (unlike number of alleles). Arlequin version 3.1 [31] was used to calculate observed heterozygosity and test for departures from Hardy-Weinberg equilibrium. The number of private alleles (i.e. alleles only found in a single population) were calculated using GDA version 1.1 [32].

To estimate parasite genetic structure among geographic areas and host species, Wright's hierarchical *F*-statistics [33] were calculated in Arlequin using an analysis of molecular variance (AMOVA) approach [31],[34]. *F_ST_* values were calculated to measure genetic differentiation among parasite populations grouped by area 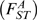 and host species 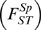, and can be defined as the correlation of alleles within these populations relative to that within the total parasite population. Inbreeding coefficients (*F_IS_* values) were also calculated, which measure the correlation of alleles within individual isolates relative to that within a defined parasite population. An important consideration when conducting population genetic analyses of parasite larval stages is that isolates sampled from a given individual host may be related by virtue of being siblings or clones. In the case of schistosomes, miracidia sampled from a definitive host may be siblings (or indeed half siblings) as a result of sexual reproduction between adult worms, while cercariae sampled from an infected snail are likely to be clones arising from asexual reproduction. *F_ST_* values among populations could therefore be inflated if the correlation of alleles within parasite infrapopulations sampled from individual hosts is not adjusted for [8] (where an infrapopulation is the population of parasites that inhabits a single individual host [35]). To minimize such potential effects when analyzing cercariae isolates, the cercariae dataset was cleared of clonal genotypes for each village, under the reasonable assumption that these would have arisen from the same snail. For miracidia, three-level hierarchical analyses were conducted such that isolates (level 1) were grouped according to infrapopulations (level 2), which were then further grouped according to geographic area or host species (level 3). Using AMOVA, genetic variance is partitioned into each level of the hierarchy, thus all *F_ST_* values calculated at level 3, i.e. among parasite populations grouped by area 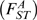 and species 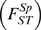, were adjusted for genetic variance components at the infrapopulation level. In order to test the potential importance of adjusting for genetic variation among parasite infrapopulations when estimating parasite genetic differentiation among groups of hosts, 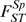 was also calculated using just two levels of hierarchy, in which isolates were simply grouped according to the host species from which they were sampled (i.e. without any subdivision of isolates according to individual host). Ninety five percent confidence intervals (95% CI) were calculated by bootstrapping over populations and subpopulations for each locus in Arlequin, thus it was possible to test whether *F_ST_* values were significantly greater than zero (which would indicate significant genetic differentiation) at the *P*<0.05 level.

Phylogenetic reconstruction was implemented using the neighbor-joining method in PowerMarker based on C.S. Chord genetic distance [36], because it has been shown by analysis of simulations to generate the correct tree topology regardless of the microsatellite mutation model [37]. Reliability of tree topology was tested by generating 100 bootstrapped trees in PowerMarker. These trees were then analyzed in Phylip version 3.67 [38] using the Consense package to calculate bootstrapping values for each cluster.

Bayesian clustering analysis was performed using STRUCTURE version 2.1 [39], a model-based program which can infer population structure without using prior information on sample origin. Simulations in STRUCTURE were carried out using a burn-in of 5 000 and a run length of 50 000, using a model in which allele frequencies were assumed to be correlated within populations. The software was run with the option of admixture, allowing for some mixed ancestry within individual isolates.

### Ethical consideration

This project was approved by the ethical boards of the Research Institute of Tropical Medicine (RITM), Brown University and Imperial College London. Village leaders were asked for consent for their village to be included in the survey, and written informed consent was obtained from all human study participants and owners of sampled domesticated animals. Personal identifiers were removed from the dataset before analyses were undertaken. The animal protocol was reviewed and approved by the Brown University Institutional Animal Care and Use Committee, the DBL-Institute for Health Research and Development (then called Danish Bilharziasis Laboratory) and the RITM's animal protection committee adhering to the institution's guidelines for animal husbandry.

## Results

### Parasite genetic structure at the definitive host level

**Figure 2:**
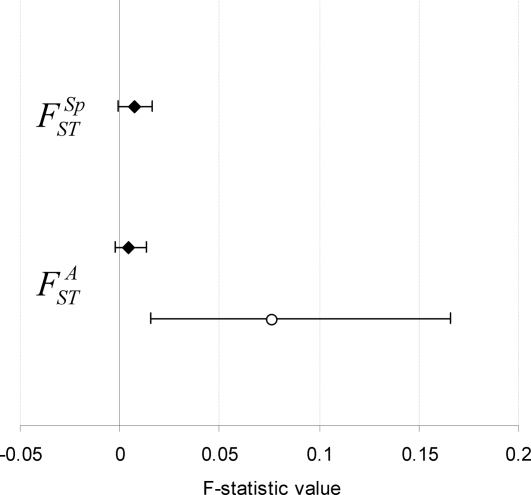
Genetic differentiation of *Schistosoma japonicum* among definitive host species 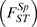 and geographic areas 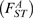. Estimates of Wright's F-statistics for genetic differentiation of *S. japonicum* miracidia (black diamonds) and cercariae (white circles) population isolates. Error bars represent 95% confidence intervals estimated from 20 000 bootstraps.

Bayesian clustering analysis using STRUCURE did not reveal any obvious clustering pattern of miracidia genotypes by geographic area within Samar (see Supporting Information, Figure S1). From hierarchical AMOVA, variation among areas was estimated to account for only 0.4% of the total genetic variation, and 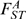 was estimated at 0.004 (95% CI from bootstrapping: −0.002 to 0.014) (Figure 2). Thus there was no evidence of significant genetic differentiation of parasite populations between the two areas at the definitive host level (although there was some evidence for this at the intermediate host level, see below). Furthermore, the upper 95% CI of 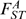 suggests that if parasite genetic differentiation does exist between the two areas, it is likely to be very small.

Given the overall lack of evidence for genetic structuring of miracidia by geographic area, isolates were divided into populations according to definitive host species regardless of the site of sampling for analyses of genetic structure across host species. Hardy-Weinberg estimates revealed high levels of genetic polymorphism within larval isolates from the four definitive host species in all seven loci, confirming the suitability of these markers for population genetics studies of *S. japonicum*. There were significant losses of heterozygosity across the majority of loci in all definitive host species (Table 2). The greatest loss of heterozygosity was observed in rat isolates, as evident from the relatively high inbreeding coefficient (*F_IS_*) for this species (Table 3), perhaps suggesting greater inbreeding of parasites within this host species. Gene diversity indices were comparable across human, dog and rat isolates (0.57–0.62), but slightly lower within pigs (0.45). Similarly, allelic richness was lowest among pig isolates (3.3) compared with population isolates from other species (4.2–4.8) (Table 3).

**Table 2:** Test for Hardy-Weinberg equilibrium.

	2AAA				J5				M5A				MF1			
	A	Ae	He	Ho	A	Ae	He	Ho	A	Ae	He	Ho	A	Ae	He	Ho
Dog	14	3	0.77	0.36^a^	12	0	0.73	0.81	6	2	0.49	0.28^a^	11	4	0.61	0.13^a^
Human	13	3	0.75	0.40^a^	12	0	0.76	0.79	4	1	0.47	0.34^a^	8	2	0.38	0.06^a^
Pig	5	1	0.45	0.20^a^	4	0	0.54	0.93	3	0	0.48	0.28^a^	3	0	0.47	0^a^
Rat	4	0	0.62	0.33^a^	9	2	0.75	0.83	3	0	0.32	0.06^a^	6	2	0.79	0.09^a^
Mean	9	1.75	0.65	0.32^a^	9.25	0.5	0.70	0.84	4	0.75	0.44	0.24^a^	7	2	0.56	0.07^a^

	MPA				RRPS				TS2				Overall			
	A	Ae	He	Ho	A	Ae	He	Ho	A	Ae	He	Ho	A	Ae	He	Ho
Dog	13	3	0.71	0.68^a^	5	1	0.55	0.56	8	2	0.50	0.19^a^	9.86	5.29	0.62	0.43^a^
Human	9	0	0.67	0.57^a^	3	0	0.55	0.53^a^	7	1	0.52	0.19^a^	8.00	4.00	0.58	0.41^a^
Pig	5	0	0.54	0.33^a^	4	1	0.49	0.43	3	0	0.37	0.15^a^	3.86	1.57	0.47	0.33^a^
Rat	8	0	0.79	0.64	4	0	0.54	0.38^a^	4	1	0.55	0.08^a^	5.43	2.86	0.62	0.35^a^
Mean	8.75	0.75	0.68	0.56^a^	4	0.5	0.53	0.48^a^	5.5	1	0.47	0.15^a^	6.79	3.43	0.58	0.38^a^

Number of alleles (A), number of private alleles (Ae), expected heterozygosity (He) and observed heterozygosity (Ho) for each microsatellite locus in *Schistosoma japonicum* isolates obtained from various definitive host species.

a Significant loss of heterozygosity (*P*<0.05).

**Table 3:** Summary statistics for *Schistosoma japonicum* population isolates across host species.

Host species	Sample size	Major allele frequency	No. of alleles	Allelic richness	PIC	Gene diversity	Ho	*F_IS_*
**Dog**	328	0.51	9.86	4.58	0.57	0.62	0.43	0.31
**Human**	409	0.54	8.00	4.15	0.53	0.58	0.41	0.30
**Pig**	42	0.68	3.86	3.25	0.40	0.45	0.33	0.30
**Rat**	42	0.54	5.43	4.82	0.56	0.57	0.35	0.45

Values are averaged across seven microsatellite loci (PIC = polymorphism information content; He = expected heterozygosity, Ho = observed heterozygosity; *F_IS_* = population-specific inbreeding coefficient).

Of the 83 different alleles observed in miracidia, 54 (65.1%) were shared across at least two host species, 18 (21.7%) were universal across all host species, and notably, the most frequent alleles at each locus were found in all host species, suggesting substantial gene-flow between parasite populations of each species. Nevertheless, 29 alleles (34.9%) were definitive host-specific, and the number of these private alleles varied at each locus and within host species, with the mean number of private alleles over all loci being highest in dogs (5.3), followed by humans (4.0), rats (2.9) and finally pigs (1.6). However, the majority of these private alleles were very rare, with 20 out of the 29 (68.9%) found at frequencies of less than 1% within their respective host species populations. Thus more private alleles may have been found in dogs and humans here simply because much higher numbers of miracidia were sampled from these species relative to rats and pigs. On the other hand, alleles 441 and 451 at locus MF1, and allele 371 at locus TS2, all of which were private to rat isolates, were observed at relatively high frequencies of 9.0%, 13.6%, and 7.7% respectively.

**Figure 3:**
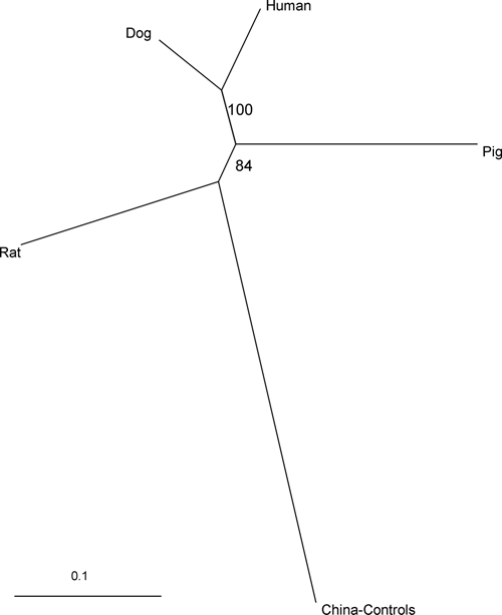
Neighbor-joining tree of *Schistosoma japonicum* miracidia from different host species in Samar, the Philippines. “China-controls” represent cercariae from Anhui Province, China. Tree is based on C.S. Chord genetic distance [35] based on 7 microsatellite loci. Bootstrap values (out of 100) are indicated at the branch points.

STRUCTURE analysis revealed no discernable clustering of genotypes according to host species, nor were any obvious clusters relating to host species observed in neighbor-joining phenograms constructed at the individual host level (data not shown). However, a phenogram at the host species level suggested that parasite genotypes sampled from dogs and humans may be more closely related to each other than with pig and rat isolates, with 100% of bootstrapped trees clustering human and dog population isolates together (Figure 3).

From hierarchical AMOVA, the within-host individual variance component, estimated here at 92%, accounted for most of the genetic diversity of miracidia samples. The within-species among-individual host variance component was estimated at just over 7%, while variation among species was estimated to account for less than 1% of the total genetic variation (Table 4). Accordingly, 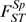 (adjusting for genetic variation among individual hosts) was estimated to be very low at 0.007, and not significant according to 95% confidence intervals estimated by bootstrapping (−0.001 to 0.016) (Figure 2).

**Table 4:** Genetic variance components for *Schistosoma japonicum* miracidia isolates at the definitive host species level.

Locus	Variance components (%)		
	Among Species	Among hosts within species	Within hosts
2AAA	1.59	9.49	88.92
J5	0.71	0.51	98.78
M5A	0.48	5.22	94.30
MF1	3.03	16.92	80.05
MPA	0.01	4.83	95.16
RRPS	−0.38	5.74	94.64
TS2	−0.92	10.54	90.38
**Overall**	**0.68**	**7.25**	**92.07**

Pairwise 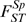 values were also calculated between parasite isolates from each host species using both two and three levels of hierarchy as described in the methods section. When using two levels of hierarchy, 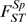 was very low (0.003) and not significant between dogs and humans (Table 5, above diagonal). All other pairwise 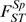 from two-level analyses were significant, with the highest value observed between rats and pigs (0.09), which supports the phenogram in Figure 3, and might suggest moderate genetic differentiation between parasite populations of these species. However, when adjusting for the individual host level in three-level analysis, all pairwise 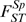 values for pigs and rats were lower and not significantly greater than 0 (Table 5, below diagonal). Pairwise 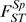 values for pigs and rats had wide confidence intervals in both two and three-level analyses, reflecting the relatively small sample sizes from these species.

**Table 5:** Pairwise 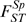 between definitive host species and snail-intermediate host *Schistosoma japonicum* isolates.

	Dog	Human	Pig	Rat	Snail
Dog	-	0.00 (0.00, 0.02)	0.04^a^ (0.01, 0.09)	0.02^a^ (0.00, 0.04)	0.01^a^ (0.00, 0.02)
Human	0.00 (0.00, 0.01)	-	0.05^a^ (0.01, 0.09)	0.04^a^ (0.00, 0.08)	0.02^a^ (0.00, 0.04)
Pig	0.02 (−0.02, 0.07)	0.02 (−0.01, 0.06)	-	0.11^a^ (0.04, 0.22)	0.03^a^ (0.02, 0.04)
Rat	0.02 (−0.01, 0.03)	0.03 (0.00, 0.07)	0.09 (0.00, 0.17)	-	0.04^a^ (0.01, 0.09)
Snail	0.00 (0.00, 0.01)	0.01 (0.00, 0.03)	−0.04 (−0.07, 0.00)	0.01 (−0.02, 0.06)	-

Values above the diagonal represent 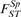 estimates not adjusting for genetic differentiation at the individual host level. Values below the diagonal represent 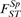 estimates adjusted for genetic differentiation at the individual host level. (95% confidence intervals shown in brackets).

a Significant genetic differentiation (*P*<0.05).

An analysis was also performed separately across human host individuals, although this did not reveal any obvious clustering of genotypes by host age, intensity of infection, occupation or sex. Genetic diversity and allelic richness indices were also comparable across these categories within humans (data not shown).

### Parasite genetic structure at the snail-intermediate host level

**Figure 4:**
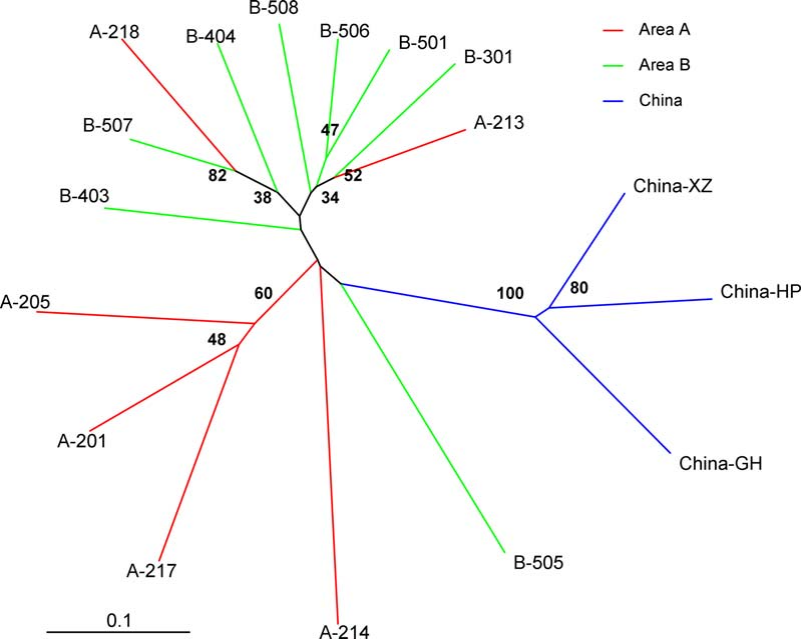
Neighbor-joining tree of *Schistosoma japonicum* cercariae from 14 villages in two geographic areas of Samar, the Philippines. Cercariae from three villages in Anhui Province, China (GH, Guanghui; HP, Heping; XZ, Xingzhuang) are also displayed. Tree is based on C.S. Chord genetic distances [35] based on 7 microsatellite loci. Geographic area and village codes are indicated at the end of each branch. Bootstrap values (out of 100) are indicated at the branch points. Branches are color coded according to geographic area.

Contrary to clustering analyses of miracidia, a neighbor-joining phenogram of village population isolates of cercariae suggested at least some degree of geographical structuring. In Figure 4, it can be seen that China cercarial population isolates clustered away from Philippines isolates in 100% of bootstrapped trees, while there also appeared to be some clustering of village isolates within the Philippines according to geographic area (although it should be noted that many nodes of this tree were not strongly supported by bootstrap analysis). Villages 201, 205, and 217 from area A formed a cluster in 60% of bootstrapped trees, while villages 501 and 506 from area B were neighbors in 47% bootstrapped trees, within a cluster in which seven out of nine villages were from area B. Nevertheless, village 218 (area A) neighbored with village 507 (area B) with strong support from bootstrap analysis (82%), and villages 213 and 301, also from different areas, were neighbors in 52% of bootstrapped trees. Genetic structure was therefore far from distinct between the two regions.

From AMOVA, variation among areas was estimated to account for 7.8% of the total genetic variation observed in cercariae, and 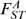 for cercariae was estimated at 0.08 (95% CI: 0.02–0.17). This suggests significant and, according to Wright's criterion, moderate genetic differentiation among areas; substantially greater than that estimated at the definitive host level (Figure 2). Even when excluding villages from which we had not obtained any miracidia, greater genetic differentiation among areas was observed in cercariae relative to miracidia (data not shown), suggesting that this was not simply a result of having obtained cercarial isolates from a wider range of villages.

When comparing the number of alleles shared between cercarial isolates and definitive host isolates, dog and human isolates had the highest number (averaged across loci) of 6.3 and 5.6 respectively, followed by rats (4.1) and then pigs (3.4). Accordingly, pairwise *F_ST_* estimates between snail population isolates and each definitive host population were lower for dogs and humans relative to pigs and rats. It is worth noting, however, that these estimates were not significant when adjusting for variation among individuals within definitive host species and among villages within cercarial isolates (Table 5).

Of the total 50 alleles found in cercarial isolates, four (8.0%) were not found in any definitive host population isolates, although all of these alleles were at relatively low frequencies (0.5–2.0%) within cercariae. Furthermore, four of these five “snail-specific” alleles were only present in cercarial isolates sampled from villages where no miracidia isolates were obtained.

## Discussion

To our knowledge this work represents the first study of the population genetic structure of *S. japonicum* across host species and geographic areas within the Philippines. The lack of genetic differentiation observed between parasite isolates from different definitive host species suggests high levels of parasite gene-flow between host species, and thus also a high frequency of *S. japonicum* transmission across species, particularly between dogs and humans. Dogs could thus potentially be a very important zoonotic reservoir of *S. japonicum* in the province of Samar, Philippines, in contrast to marshland regions of China where parasite genotypes from humans have been demonstrated to cluster with bovines and away from other domesticated animals such as dogs, cats, pigs and goats [16].

This molecular result is consistent with parasitological survey data from the same region of the Philippines, which found a mean prevalence across 50 villages of 14.9% among dogs, the highest detected across all domesticated animal species sampled, with prevalence reaching up to 86.3% in some villages. In comparison, the mean prevalence observed in cats, pigs and water buffalo was much lower at less than 2%, with only rats showing a higher mean prevalence (29.5%) than dogs [4]. The intensity of infection in dogs was also the highest among domesticated animals [5]. Moreover, levels of infection among humans were observed to be significantly associated with the mean intensity of infection among dogs, with each unit increase in the village-level mean eggs per gram associated with a 4% increase in the prevalence of infection in humans [5]. Dogs are owned by a high proportion of households in rural Philippine communities and usually permitted to roam freely, often even entering or feeding in other household premises as they scavenge for food [40],[41]. Such behavior might be expected to facilitate environmental contamination by *S. japonicum*-infected dogs in areas overlapping with human activity. Furthermore, census data from our study villages show a mean number of 104.9 dogs per village, which is almost three times that of water buffalo (36.2) and somewhat larger than the number of cats (90.4) [4]. Theoretical models of multi-host parasite evolution by Gandon [42] suggest that it may be adaptive to evolve higher transmission to host types that are more abundant, so it seems reasonable to hypothesize that *S. japonicum* in Samar may have evolved to exploit dogs as opposed to bovines as they offer greater transmission potential. Studies which aim to elucidate, for example, relationships between cercarial exposure and subsequent levels of infection in different host species could help elucidate whether this is the case.

Despite the relatively low sample sizes obtained from rats and pigs, private alleles were observed in parasite isolates from each of these species, which, combined with the measures of genetic differentiation and a phenogram at the species level, suggests that there may be less gene-flow between parasite populations of these species and humans relative to that between dogs and humans. This is consistent with the observation that village-level infection intensities in these animals were not significantly associated with those in humans [5]. Nevertheless, the molecular results with regard to rats and pigs should be interpreted with some caution, as evident from the wide confidence intervals for *F_ST_* values among parasite populations of these species, highlighting a need for future studies with increased sample sizes of these animals. It should also be noted that rats and pigs were sampled from just one area each, although the fact that there appears to be very little, if any, genetic differentiation between the two areas suggest that geographic confounding may not have been particularly important here. In a recent mathematical transmission dynamics model, the prevalence of infection in rats was suspected to contribute somewhat to prevalence of infection in humans [6]. Thus the role of rats in particular in zoonotic transmission of *S. japonicum* in Samar remains unclear.

The fact that *F_ST_* values between parasite isolates of different species lost significance when adjusting for individual host level provides empirical evidence for the importance of adjusting for individual host level when sampling larval stages of multi-host parasites, otherwise the relatedness of offspring from adult parasite infrapopulations within host individuals will inflate *F_ST_* values among species [8].

Parasite isolates from pigs showed the lowest genetic diversity, not only in measures of expected heterozygosity, but also in terms of allelic richness which takes into account the size of the sample, suggesting this result may not simply have been due the relatively small number of isolates from this species. Pigs are often, but not always, penned or tethered, and thus might be exposed to a smaller, more localized parasite gene pool. An alternative, not mutually exclusive, explanation could be that the pig isolates represent longer term infections with subsequent reduced diversity, if pigs are exposed as free-roaming young piglets before being penned or tethered at adulthood. Furthermore, studies in China have shown pigs to be less susceptible to infection (at least with the Chinese ‘strain’ of *S. japonicum*), with low rates of worm establishment compared to other mammalian hosts [4], thus the reduced diversity could reflect bottlenecking of genotypes during the infection process in pigs.

In terms of geographic structuring, it is interesting that greater and more significant genetic differentiation among areas was observed at the snail-intermediate host level relative to the definitive host level. One reason for this could be that definitive hosts, particularly humans, are more mobile and longer-lived, and thus have greater potential to acquire infections from various geographic areas. Snails on the other hand could be more localized, and are certainly shorter lived; thus snail colonies within a given geographic area may acquire infections from a smaller, more localized pool of infected definitive-host individuals. In light of this hypothesis, it would be interesting to see whether, under any increasing drug administration in the Philippines, parasite genotypes in definitive hosts (particularly humans) become more geographically clustered due to the fact that infections have not accumulated from such a range of geographic areas.

Stronger geographic structuring of *S. japonicum* at the intermediate host level could also be due to differential compatibility of parasite genotypes with allopatric and sympatric snail genotypes. Previous studies suggest that strain-specificity for schistosomiasis tends to be stronger at the snail stage compared to the definitive host stage [43],[44], which may result in more selective uptake and/or output of parasite genotypes, and hence more structuring. A study by Hope and McManus [43] however, found no genetic variation between *O. quadrasi* snails from different regions of the Philippines. Indeed, this could explain why geographic structuring and diversity of *S. japonicum* in the Philippines appears to be far less distinct than observed in China [17], where two different *O. hupensis* subspecies exist (*O. h. robertsoni* in mountainous regions and *O. h. hupensis* in lake/marshland regions), and even populations within these subspecies show marked genetic variation [45],[46],[47]. It is worth noting, however, that these previous studies on the genetic diversity of *Oncomelania* populations are largely based on restriction fragment length polymorphism markers; thus future microsatellite studies of sympatric snail populations may be important to pursue in order to obtain molecular results more comparable with those on *S. japonicum*.

Less distinctive geographic structuring of *S. japonicum* in Samar, relative to China, could also reflect frequent import and export of infections between geographic areas. Thus it may be inappropriate to assume that transmission cycles within villages are largely self-contained, raising important implications for control interventions, which may be more effective if implemented simultaneously across all villages and regions. Samar Province in the Philippines has a very wet climate throughout the year, which could facilitate substantial mixing of snail populations and year-round transmission across interconnected watersheds, in contrast to *S. japonicum*-endemic regions in China where the climate, and thus also transmission, is much more seasonal [48],[49]. This phenomenon could also explain why genetic structuring of *S. japonicum* across definitive host species, which was not evident in the present Philippines study, has been observed in China, both in a study by Wang et al. [16] and in an ongoing study within our group, where more “boom and bust” snail population dynamics could force more sub-structuring in transmission. Indeed, one could even speculate that such factors have led to different evolutionary strategies of *S. japonicum* in the two countries, with the Philippines parasite having evolved a more generalist strategy, with genotypes displaying comparable transmission potentials across several definitive host species. In China, on the other hand, *S. japonicum*, while still displaying a generalist strategy in that it can infect a range of host species, may have achieved this by evolving into multiple specialist “strains” which show differential fitness depending on definitive and/or intermediate host types, perhaps resulting from and/or resulting in greater structuring of transmission according to host species and geographic area.

To conclude, this study suggests there is frequent transmission of *S. japonicum* across different mammalian host species, and perhaps also across geographic areas, in Samar province of the Philippines, with dogs potentially playing a highly important role in zoonotic transmission. These findings raise important applied concerns for current chemotherapy-based control programs, which may be inefficient if humans are rapidly re-infected by animal host reservoirs. Indeed, unpublished data from the STEP study suggests that the risk of re-infection was approximately 11% over 12 months. In addition, the recent anti-schistosomal mass treatment campaign in Samar resulted in far lower participation than desired [50]. It may therefore be crucial for control efforts to include animals such as dogs, for example via chemotherapy and population control, if transmission control is to be achieved, although the feasibility of such measures is uncertain at this stage. Finally, in contrast to previous evidence suggestive of a potential “strain-complex” of *S. japonicum* in China relating to different definitive host species, this study did not reveal any host-related differentiation of *S. japonicum* in the Philippines, raising theoretical implications concerning the evolution of multi-host pathogens and how this may vary even within a single species of pathogen.

## Supporting Information

Figure S1 Bayesian clustering analysis of *Schistosoma japonicum* miracidia genotypes using STRUCTURE. Each bar represents a single isolate, and isolates are grouped along the x-axis according to geographic area. The y-axis represents the probability of each isolate belonging to each of two assumed clusters (color coded in red and green). The figure indicates a lack of genetic structure between areas A and B.
(3.27 MB TIF)

Alternative Language Abstract S1 Translation of the Abstract into Chinese by Da-Bing Lu
(0.13 MB PDF)

Alternative Language Abstract S2 Translation of the Abstract into French by Hélène Carabin
(0.06 MB PDF)

Alternative Language Abstract S3 Translation of the Abstract into Spanish by María-Gloria Basáñez
(0.01 MB PDF)
