# Supplementary figures and images for: Population Genetics of Schistosoma japonicum within the Philippines Suggest High Levels of Transmission between Humans and Dogs
Source: PLoS Negl Trop Dis. 2008 Nov 25;2(11):e340. doi: 10.1371/journal.pntd.0000340 (PMC2582952; doi:10.1371/journal.pntd.0000340)

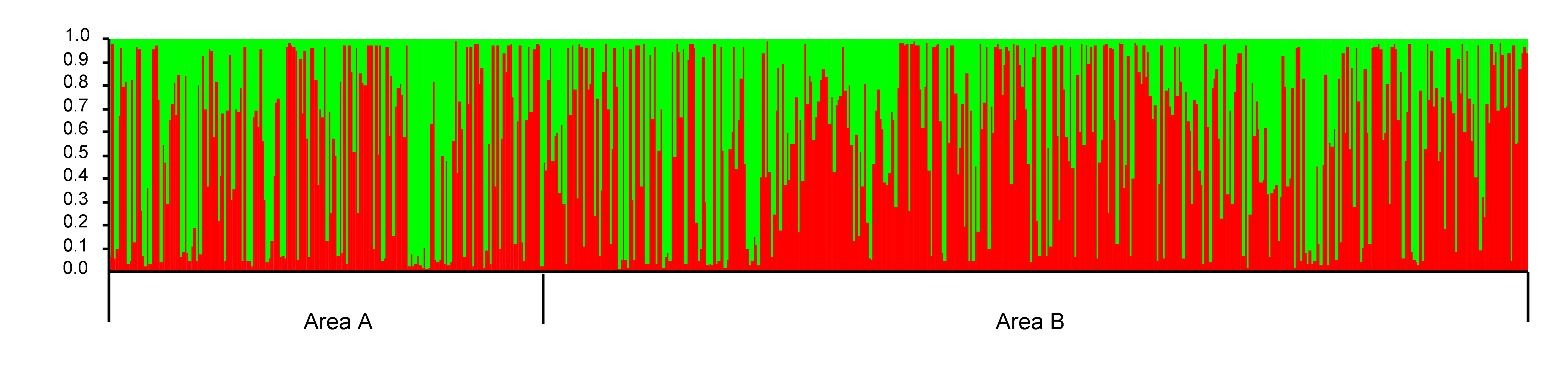

Supplement: Figure S1 — Bayesian clustering analysis of Schistosoma japonicum miracidia genotypes using STRUCTURE. Each bar represents a single isolate, and isolates are grouped along the x-axis according to geographic area. The y-axis represents the probability of each isolate belonging to each of two assumed clusters (color coded in red and green). The figure indicates a lack of genetic structure between areas A and B. (3.27 MB TIF) [file pntd.0000340.s001.tif]
