# Supplementary material for: Population Genetics of Schistosoma japonicum within the Philippines Suggest High Levels of Transmission between Humans and Dogs
Source: PLoS Negl Trop Dis. 2008 Nov 25;2(11):e340. doi: 10.1371/journal.pntd.0000340 (PMC2582952; doi:10.1371/journal.pntd.0000340)
Supplement: Alternative Language Abstract S1 — Translation of the Abstract into Chinese by Da-Bing Lu (0.13 MB PDF) [file pntd.0000340.s002.pdf]

Chinese translation of Abstract provided by Da-Bing Lu

背景：日本血吸虫是唯一的动物性传播具有重要意义的血吸虫，目前在菲律宾和中国仍为一重要公共卫生问题。在中国部分流行区耕牛被疑作重要传染源，但在菲律宾除人外各种哺乳动物在其传播中的相对地位尚待评定。因此，我们在菲律宾开展了日本血吸虫种群基因研究，旨在描述其在各种宿主间以及地区间传播方式。

方法和重要发现：在菲律宾 Samar 省 2 个试区，收集人、狗、猪和鼠的粪便，孵化收集日本血吸虫毛蚴；对中间宿主钉螺进行逸蚴，收集尾蚴；实验室内采用 7 个微卫星基因位点分析各群蚴虫标本基因型。推算的血吸虫种群间遗传距离（F_{ST}）和种群系统发生树提示各血吸虫终宿主间，尤其是狗和人间有着高度基因流。尽管尾蚴标本显示一定程度种群地区间差异，但源于终宿主血吸虫毛蚴标本则表明无明显种群地区间差异，后者可能归究于流行区间频繁的感染输出与输入。

结论与意义：研究表明日本血吸虫在各终宿主间高度传播，并提示在制定控制项目时应考虑狗在传播中作用。同时，当一地区人群流动频繁时建议采取地区性化疗项目。
