# Supplementary material for: Population Genetics of Schistosoma japonicum within the Philippines Suggest High Levels of Transmission between Humans and Dogs
Source: PLoS Negl Trop Dis. 2008 Nov 25;2(11):e340. doi: 10.1371/journal.pntd.0000340 (PMC2582952; doi:10.1371/journal.pntd.0000340)
Supplement: Alternative Language Abstract S2 — Translation of the Abstract into French by Hélène Carabin (0.06 MB PDF) [file pntd.0000340.s003.pdf]

French translation of Abstract provided by Hélène Carabin

Résumé

Information de base: *Schistosoma japonicum*, qui demeure un problem de santé publique considérable aux Philippines et en Chine, est la seule espèce de schistosome pour laquelle une transmission zoonotique existe. Alors qu'il est bien établi que les bovins constituent un réservoir d'infection important en China, le rôle relatif joué par différents hôtes mammifères non-humains dans la transmission de l'infection aux humains est inconnu aux Philippines. Afin de déterminer le patron de transmission entre espèces et zones géographiques, nous avons examiné la génétique des populations de *S. japonicum* aux Philippines.

Méthodes/principaux résultats: Les miracidies de *S. japonicum* (éclos d'œufs présents dans les selles) provenant d'échantillons humains, canins, porcins et de rats ainsi que les cercariae produits par les escargots hôtes intermédiaires, ont été recueillis dans deux zones géographiques de la province de Samar. Chaque isolat a été examiné à l'aide de sept locus microsatellites multiplexés. Les valeurs F_{ST} de Wright et les arbres phylogéniques calculés pour les populations de parasites indiquent qu'il existe une fréquence élevée de flux génétique entre les hôtes définitifs, et tout particulièrement entre les chiens et les humains. La différentiation génétique entre les zones géographiques n'était pas importante au niveau de l'hôte définitif, ce qui laisse supposer un flot important d'import et d'export entre villages, malgré la présence de structure géographique au niveau de l'escargot-hôte intermédiaire.

Conclusion/signification: Ces résultats indiquent qu'il existe de très hauts niveaux de transmission entre les espèces hôtes, et laisse supporter que le rôle que jouent les chiens dans la transmission devrait être pris en compte lors de la planification de programmes de contrôle. De plus, un programme de traitement régional est recommandé dans les cas où la migration humaine est considérable.
