# Supplementary material for: Population Genetics of Schistosoma japonicum within the Philippines Suggest High Levels of Transmission between Humans and Dogs
Source: PLoS Negl Trop Dis. 2008 Nov 25;2(11):e340. doi: 10.1371/journal.pntd.0000340 (PMC2582952; doi:10.1371/journal.pntd.0000340)
Supplement: Alternative Language Abstract S3 — Translation of the Abstract into Spanish by María-Gloria Basáñez (0.01 MB PDF) [file pntd.0000340.s004.pdf]

Spanish translation of Abstract provided by María-Gloria Basáñez

Resumen

Antecedentes: *Schistosoma japonicum*, la única especie de esquistosoma para la cual se considera que la transmisión de origen zoonótico hacia los humanos es de suma importancia, continúa representando un grave problema de salud pública en las islas Filipinas y en China continental. Mientras en ciertas partes de China se sospecha que los bovinos constituyen los principales reservorios zoonóticos, en Filipinas aún no se ha determinado la contribución relativa de los mamíferos no humanos a la transmisión. Por consiguiente, hemos examinado la genética de poblaciones de *S. japonicum* en Filipinas a fin de dilucidar posibles patrones de transmisión entre varias especies de hospedadores y áreas geográficas.

Metodología / Hallazgos Principales: En dos áreas geográficas de la Provincia de Samar, se colectaron muestras de miracidios de *S. japonicum* obtenidas al eclosionar huevos del parásito hallados en muestras fecales derivadas de humanos, perros, cerdos, y ratas, así como cercarias obtenidas a partir de caracoles hospederos intermediarios. El genotipo de los aislados individuales fue determinado mediante el uso de siete loci de microsatélites. Los valores de F_{ST} de Wright y los árboles filogenéticos calculados para las poblaciones de parásitos sugieren frecuencias elevadas de flujo génico entre las especies de hospederos definitivos estudiadas, particularmente entre perros y humanos. No se encontró evidencia de diferenciación genética entre parásitos de las dos áreas geográficas estudiadas a nivel de hospederos definitivos, lo que sugiere importación y exportación frecuentes de infecciones entre poblados. Ello no obstante, se halló cierta evidencia de estructuración geográfica de parásitos a nivel del caracol.

Conclusiones / Significación: Estos resultados sugieren elevados niveles de transmisión entre especies e indican la importancia de considerar el rol de los perros a la hora de planificar programas de control, así como el implementar estrategias regionales de tratamiento donde la migración humana es extensa.
